# CD8+ T Cells in GCA and GPA: Bystanders or Active Contributors?

**DOI:** 10.3389/fimmu.2021.654109

**Published:** 2021-03-18

**Authors:** Rosanne D. Reitsema, Annemieke M. H. Boots, Kornelis S. M. van der Geest, Maria Sandovici, Peter Heeringa, Elisabeth Brouwer

**Affiliations:** ^1^ Department of Rheumatology and Clinical Immunology, University of Groningen, University Medical Center Groningen, Groningen, Netherlands; ^2^ Department of Pathology and Medical Biology, University of Groningen, University Medical Center Groningen, Groningen, Netherlands

**Keywords:** giant cell arteritis, granulomatosis with polyangiitis, CD8+ T cells, vasculitis, aging

## Abstract

Vasculitis refers to inflammation of blood vessels and can cause a variety of serious complications depending on which vessels are affected. Two different forms of vasculitis are Giant Cell Arteritis (GCA) and Granulomatosis with Polyangiitis (GPA). GCA is the most common form of vasculitis in adults affecting the large arteries and can lead to visual impairment and development of aneurysms. GPA affects small- and medium-sized blood vessels predominantly in the lungs and kidneys resulting in organ failure. Both diseases can potentially be fatal. Although the pathogenesis of GCA and GPA are incompletely understood, a prominent role for CD4+ T cells has been implicated in both diseases. More recently, the role of CD8+ T cells has gained renewed interest. CD8+ T cells are important players in the adaptive immune response against intracellular microorganisms. After a general introduction on the different forms of vasculitis and their association with infections and CD8+ T cells, we review the current knowledge on CD8+ T-cell involvement in the immunopathogenesis of GCA and GPA focusing on phenotypic and functional features of circulating and lesional CD8+ T cells. Furthermore, we discuss to which extent aging is associated with CD8+ T-cell phenotype and function in GCA and GPA.

## Introduction

The vasculitides are a heterogeneous group of disorders characterized by inflammation of blood vessels. Classification of the different forms of primary vasculitis is defined by the 2012 International Chapel Hill Consensus conference (1) and is based primarily on the size of the inflamed vessels. The distinct forms of vasculitis also differ from each other regarding age of onset, genetic predisposition, pathogenesis and affected organs.

The onset of some forms of vasculitis has been linked to infectious triggers. A prime example is granulomatosis with polyangiitis (GPA), which has been associated with various microbial agents, in particular *Staphylococcus aureus.* GPA is a severe systemic autoimmune disease that predominantly affects the elderly. The disease is characterized by necrotizing vasculitis of small- to medium-sized blood vessels and the presence of anti-neutrophil cytoplasmic antibodies (ANCA) mainly directed against proteinase 3 (PR3). Due to inflammation of small blood vessels, several organs and tissues can be severely affected. In GPA, especially upper and lower respiratory tract and kidney involvement are common. Besides necrotizing vasculitis, GPA is characterized by granulomatous inflammation of the respiratory tract. Studies have shown that the majority of GPA patients are nasal carriers of *S. aureus* which correlated with higher relapse rates although a direct link between *S. aureus* carriage and disease activity or relapse risk remains to be established (2, 3). Moreover, in a subset of GPA patients a clinical benefit from treatment with antibiotics has been demonstrated adding to the notion that a microbial factor may trigger the disease (4).

Giant cell arteritis (GCA) is the most common form of large vessel vasculitis and affects females twice as often as men. GCA is strongly age-related as it only affects people older than 50 years of age (5). The symptoms experienced by GCA patients are largely dependent on the anatomic localization and type of the affected vessels. Different studies reported an association between disease onset and infections with specific pathogens such as *Mycoplasma pneumoniae*, Parvovirus B19, Herpes Zoster and Parainfluenza virus but none of these associations could be conclusively validated in follow-up studies (6–8). This does not exclude a role for infections in the onset of GCA, but rather suggests the involvement of shared inflammatory pathways that can be activated by various infectious agents.

Other forms of vasculitis in which infections have been strongly implicated include Kawasaki disease (KD) (9–11), a medium vessel vasculitis affecting young Asian populations, Polyarteritis nodosa (PAN), a medium-sized vessel vasculitis affecting adults (12–15) and Takayasu Arteritis (TA), which affects younger adults from Asian populations (16).

Our immune system is designed to protect our body against infectious agents and utilizes specialized immune cells for this purpose. CD8+ T cells are key in the anti-viral defense by clearance of virus-infected cells, but also act against intracellular bacterial infections. It is therefore not surprising that the role of CD8+ T cells has been investigated in several forms of vasculitis that have been associated with infectious agents. The forms of vasculitis in which the role of CD8+ T cells is most pronounced, are diseases affecting children (KD) or younger adults (TA) (summarized in **Table 1**). CD8+ T cells are highly prevalent in inflammatory infiltrates of KD patients and upregulated CD8+ and interferon pathway genes were detected in post mortem coronary artery biopsies (17, 18). In TA CD8+ T cells are also abundantly present in affected arteries and perforin was suggested to induce vascular cell injury (25). Regarding older adults with PAN, available data is limited to a small number of case series, which revealed the presence of CD8+ T cells at the site of vascular inflammation, mostly outnumbering the CD4+ T cells (29–32).

**Table 1:** Evidence suggesting CD8+ T cell involvement in Kawasaki disease and Takayasu Arteritis.

Kawasaki disease	Takayasu Arteritis
* Strongly associated with (viral) infections (9–11). * Transmural infiltration of more memory CD8+ T cells than CD4+ T cells in biopsies of coronary artery aneurysms (17). * Genes related to CD8+ T-cell activation and type 1 interferon induced genes are upregulated in biopsies (18). * CD8+ T cells, but not CD4+ T cells, are required for KD development in a murine model of KD (19). * The frequency of activated CD8+ T cells, defined by HLA-DR expression, was higher in peripheral blood of active KD patients compared to controls (20). * Treatment with intravenous immunoglobulin (IVIG) inhibits CD8+ T-cell activation (20). * Patients that were IVIG resistant had a higher percentage of peripheral blood CD8+ HLA-DR+ T cells compared to responders (20). * Percentages of the costimulatory receptor NKG2D-expressing CD8+ T cells were lower in peripheral blood of acute KD than in HCs (21). NKG2D-expressing CD8+ T cells could also have migrated to tissues or NKG2D could be downregulated in response to ligand-bound activation.	* MHC-I genes, especially HLA-B52 is strongly associated with TA, suggesting involvement of CD8+ T cells (22–24). * CD8+ T cells are present in aortic tissues (25, 26). * A study in a small group of rather old TA patients suggests that TA aorta biopsies have more infiltrating CD8+ T cells than temporal artery biopsies of GCA patients (26). * Perforin was expressed in CD8+ T cells in aortic tissues of TA patients, which was suggested to induce vascular cell injury (25) * 248 genes in CD4+ and 432 genes in CD8+ T-cell samples differed between TA and HCs. TA patients had upregulation of type I interferon genes (27) * Strong expression of NKG2D, a costimulatory receptor for CD8+ T cells and NK cells, and its ligand MICA in aortic lesions of TA patients (28).

Although disease onset and progression in GCA and GPA have also been linked to infectious agents, the contribution of CD8+ T cells to the pathogenesis of these diseases is largely unknown. Studies on immune mechanisms involved in disease pathogenesis have mainly focused on the roles of macrophages and CD4+ T cells in GCA, and additionally on neutrophils and autoantibody producing B cells in GPA. Recent studies however, have clearly demonstrated the presence of CD8+ T cells in vasculitis lesions in both GCA and GPA (33, 34). We deemed this observation of particular interest due to the possible role of infectious triggers in both diseases and the notion that aging, having a profound influence on especially CD8+ T-cell functions, presents a risk factor for both GCA and GPA.

Therefore, we here review and discuss the studies on CD8+ T-cell involvement in the pathogenesis of GCA and GPA to determine whether CD8+ T cells are active contributors to disease pathogenesis or just bystanders with limited pathogenic functions, and to determine to which extent aging affects the function and phenotype of CD8+ T cells in GCA and GPA. After a general overview of CD8+ T-cell function in health, disease and aging we discuss the current knowledge on CD8+ T cells in GCA and GPA with respect to circulating and lesional phenotypes, transcriptomic profiles and function. Paired medical subject (MeSH) headings used for our literature search included giant cell arteritis, temporal arteritis, granulomatosis with polyangiitis, Wegener’s, CD8+ T cells and ANCA-associated vasculitis. The reference lists of the articles selected with these keywords were also used to include additional relevant articles. Single case reports, reports in <5 patients, studies without any clear patient characteristics, and inaccessible whole text of studies were excluded.

## CD8+ T Cells in Health and Disease; Activation and Subset Differentiation

The primary function of CD8+ T cells is to identify and eliminate virus- or bacteria-infected, malignant and damaged cells. Upon recognition of their cognate peptide presented by MHC class I, CD8+ T cells can kill their target cells via perforin and granzymes and release cytokines such as Interferon (IFN)-γ and tumor necrosis factor alpha (TNF-α). Some cytokines are directly cytotoxic, but others recruit and activate other effector cells. Fas-Fas-Ligand interactions between target cells and CD8+ T cells, respectively, result in death of target cells as well.

Next to these protective functions, CD8+ T cells can also be detrimental and contribute to autoimmune diseases. For instance, auto-reactive CD8+ T cells seem to contribute to autoimmune pathology in type 1 diabetes, alopecia areata, multiple sclerosis and inflammatory bowel disease (35–38). In healthy individuals, several mechanisms are in place that prevent the activation of CD8+ T cells upon autoantigen presentation. In patients with an autoimmune disease however, failing regulatory mechanisms and other factors can result in activation of autoreactive CD8+ T cells. These factors include among others, the activation state of the dendritic cells (DCs), the number of antigen-MHC complexes, TCR affinity, the local cytokine milieu and the functionality of regulatory CD4+ and CD8+ T cells (38).

**Figure 1:**
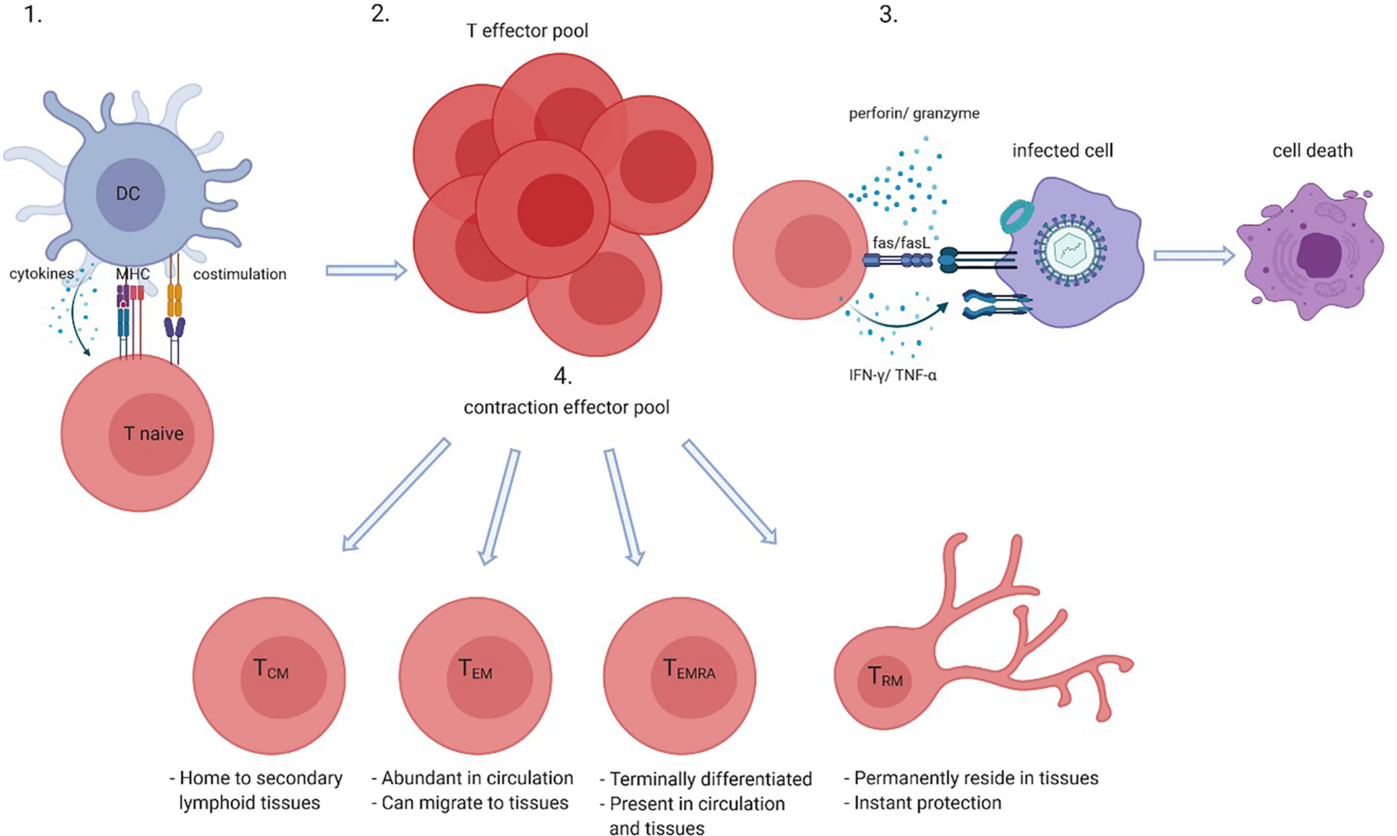
CD8+ T-cell activation and differentiation. Naive CD8+ T cells when presented with their cognate antigen by antigen presenting cells such as dendritic cells (DCs) in the context of co-stimulation (CD28-CD80/86) and in the presence of cytokines become activated (1) which gives rise to an antigen-specific CD8+ effector pool (2). Effector CD8+ T cells kill infected, tumor or damaged cells by perforin and granzyme, Fas and Fas ligand (FasL) interactions and directly or indirectly via cytokines such as interferon (IFN)-γ and tumor necrosis factor (TNF)- α (3). After antigen clearance most effector cells go into apoptosis which leads to contraction of the effector pool, but long-lived memory cells such as central memory (T_CM_), effector memory (T_EM_), memory cells that re-express CD45RA T_EMRA_, and tissue residing memory (T_RM_) cells survive (4). Created with BioRender.com.

Activation of CD8+ T cells, in either the context of health or disease, leads to a sequence of events that results in differentiation of naive CD8+ T cells into memory cells and thus the generation of a pool of antigen-specific memory CD8+ T cells (**Figure 1**). Upon proper antigen presentation and appropriate co-stimulation and cytokine involvement, naive CD8+ T cells proliferate and give rise to clonal expansions of antigen-specific effector CD8+ T cells. Contraction of the effector pool takes place after antigen clearance via apoptosis of most short-lived effector cells. Long-term memory is generated by the survival of a small subset of long-lived antigen-specific memory CD8+ T cells. The memory CD8+ T cell pool generated in response to antigen recognition consists of several subsets. Central memory CD8+ T cells (T_CM_) are hardly found in the circulation as these home to secondary lymphoid organs whereas effector memory CD8+ T cells (T_EM_) cells are more abundant in the circulation and often migrate to the (inflamed) tissues (39, 40). Nowadays it is clear that tissue residing memory CD8+ T cells (T_RM_) have a role in instant protection of the host tissues to both internal and external imminent threats. Indeed, CD8+ T_RM_ cells are thought to reside in the tissues permanently and do not seem to circulate. Antigen recognition by T_RM_ cells also promotes the recruitment and activation of other innate and adaptive immune cells to the tissue. Another memory CD8+ T-cell subset detected in both the circulation and the (inflamed) tissues is the T_EMRA_ subset. T_EMRA_ cells are late-stage memory CD8+ T cells that re-express CD45RA and are likely terminally differentiated (40). The latter subset is characteristic of aging and latent CMV infection.

## CD8+ T Cells, Aging, CMV and Inflammaging

**Figure 2:**
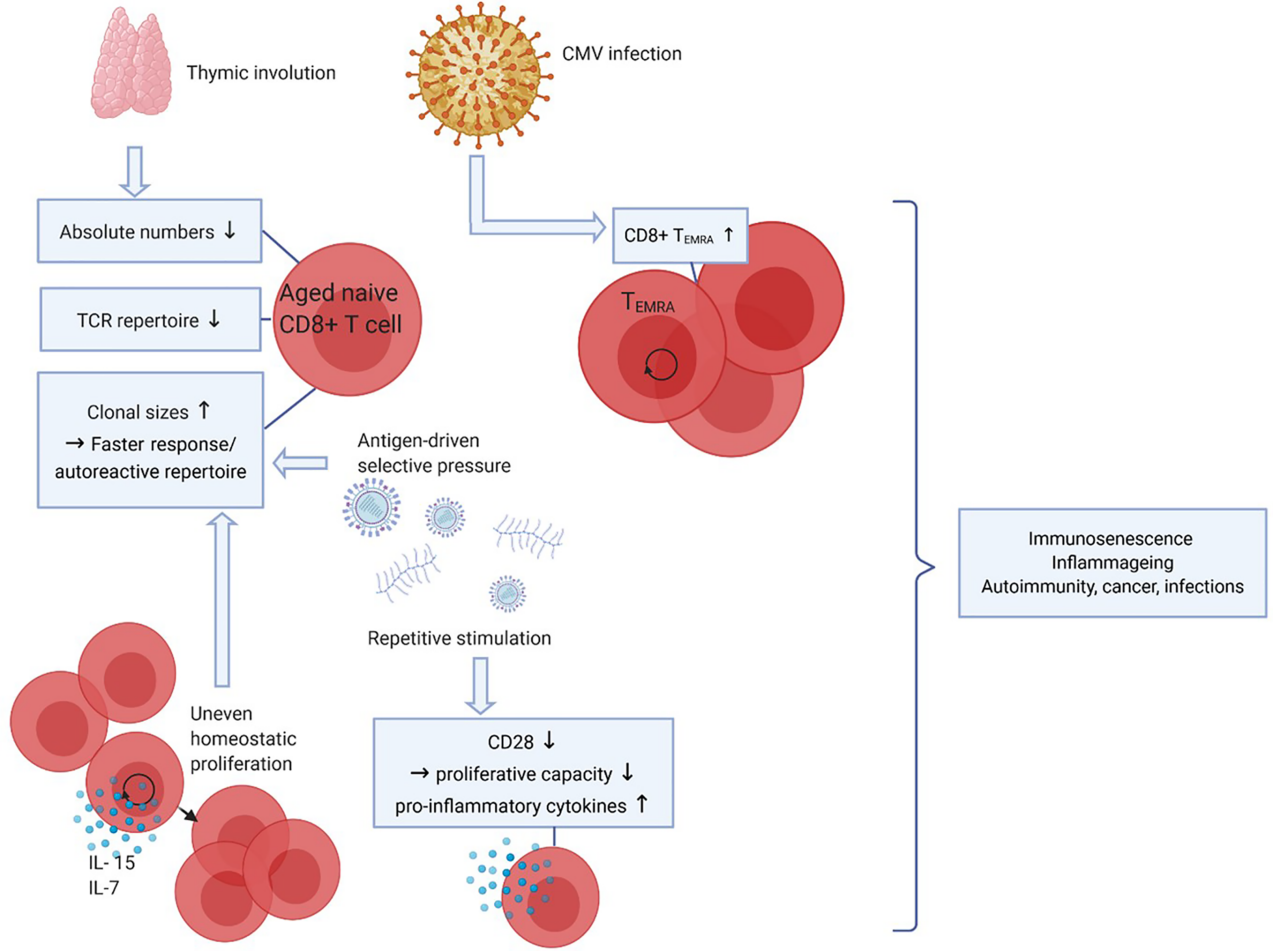
Effects of aging on CD8+ T cells. Upon aging, the thymus size decreases which leads to a reduced output of naive CD8+ T cells, thereby affecting the diversity of the T-cell receptor (TCR) repertoire. Also, due to antigen-driven selective pressure and uneven homeostatic proliferation naive clonal sizes increase, which leads to a faster response to certain antigens but may also favor an autoreactive repertoire. Repetitive stimulation by (e.g. latent viral) antigens decreases CD28 expression, CD8+ T cells lacking CD28 have decreased proliferative capacity but produce more pro-inflammatory cytokines. CMV infection leads to increasing CD8+ T_EMRA_ cell numbers. Together, these phenomena lead to decreased immune cell functions (immunosenescence), low-grade inflammation (inflammaging) and a higher risk to develop autoimmunity, cancer and infections. Created with BioRender.com.

Upon aging, many changes in the immune system occur, but the decline of naive CD8+ T-cell numbers is the most profound hallmark of aging (**Figure 2**). The thymus involutes early in life around puberty, and T-cell homeostatic proliferation of naive T cells is necessary to maintain the pool of naive T cells. This process causes absolute numbers of naive CD4+ T cells to remain stable in elderly but naive CD8+ T-cell numbers still profoundly decrease upon aging (41–43). In both CD4+ and CD8+ naive T cells the size of the TCR repertoire declines between the ages of 30 and 70 years. Although this may limit the size of the TCR repertoire, the TCR repertoire still remains very large and the decrease in diversity in elderly is predicted to unlikely have functional consequences (44, 45).

Aging is often associated with T-cell clonal expansions especially in the naive repertoire. Clonal sizes increase due to antigen-driven selective pressures or by uneven homeostatic proliferation in response to homeostatic cytokines. The latter seems to be the cause of increased clonal sizes within both the CD4+ and the CD8+ naive pools, as the clonally expanded naive CD8 T cells had different TCR sequences compared to memory CD8+ T cells. Increases in clonal sizes within the naive T-cell pool upon aging might be beneficial for the host as expanded clones may result in a faster response to antigenic challenges (44, 45). Conversely, this may also generate an auto-reactive repertoire and contribute to development of autoimmune diseases with aging.

Latent infections with viruses such as varicella zoster virus, Epstein-barr virus (EBV) and CMV are well known to have an impact on the T-cell composition in the blood. This is likely caused by continuous stimulation of the immune system. As CMV is a relatively large virus expressing many proteins, the CD8+ T-cell immune repertoire controlling the virus is quite broad. CMV seropositivity is well known to increase with age and 50-85% of adults of 40 years and older are seropositive (46). As a substantial repertoire of CD8+ T cells needs to control this infection the development and expansion of highly differentiated CD8+ T_EMRA_ cells have been documented (42, 47). As a result CD8+ T_EMRA_ cells accumulate with age, a progress called memory inflation (48, 49). This process is kept in balance to some extent because CD8+ T_EMRA_ cells have a lower proliferative capacity. At the same time, CD8+ T_EMRA_ cells are highly cytotoxic and can produce many cytokines, which is beneficial in combatting infections, and thus may compensate for their lower proliferative potential (45).

Another important marker of T-cell aging and of highly differentiated cells such as CD8+ T_EMRA_ cells is the lack of CD28 expression. CD28 is the most important co-stimulatory receptor expressed by T cells as it interacts with CD80/86 on professional antigen presenting cells. Repetitive stimulation of CD8+ T cells, however, leads to downregulation of CD28 (50). CD8+CD28- cells are highly differentiated, produce pro-inflammatory factors and proliferate poorly to TCR stimulation but are still able to proliferate in response to cytokines like interleukin (IL)-15 (51). As senescent cells are unable to proliferate in response to any signal, CD8+CD28- cells are not considered truly senescent but instead described as “senescent-like”. The higher proportion of CD8+CD28- cells in aged adults has been linked to decreased responses to infectious agents and vaccines (52).

Clonally expanded naive CD8+ T cells and accumulation of highly differentiated CD8+ T cells with high cytotoxic capacity are normal phenomena that are related to aging. However, these phenomena alongside decreased function of other immune cells such as B cells can lead to decreased immune clearance and lower ability to maintain self-tolerance. Immunosenescence is a general term used to describe the waning of immune functions with aging. Immunosenescence may lead to increased occurrence of infections, cancer and autoimmunity. Furthermore, high cytokine production, for instance by differentiated CD8+ T cells, can lead to a state of low-grade inflammation in the elderly, also known as inflammaging (53). Immunosenescence and inflammaging have been associated with several autoimmune diseases, such as systemic lupus erythematosus (SLE) and rheumatoid arthritis (RA) (54, 55).

## Pathogenesis of GCA

GCA is a granulomatous vasculitis that can affect the aorta and its proximal branches, including cranial vessels. Symptoms include severe headaches, scalp tenderness or necrosis, visual disturbances or even visual loss (56). Most GCA patients have a relapsing disease course. Studies indicated that human leukocyte antigen (HLA) class II genes, in particular HLA-DRB1*04, are associated with disease susceptibility, visual loss and glucocorticoid resistance (57–59), HLA class I genes have been associated with genetic susceptibility as well (57, 60).

It is unclear what causes the disease although several infectious agents have been proposed to be involved in triggering the immune response that results in severe inflammation of the vessel wall. However, an unknown endogenous factor (e.g. danger associated molecular pattern (DAMP)) should not be excluded and may function as a trigger for initiation of the disease in a susceptible host. Activation of tissue-residing dendritic cells (DCs) bearing toll-like receptors (TLRs) in the adventitia of the vessel wall by such an unknown trigger may start the cascade of disease-specific inflammatory processes.

The proposed sequences of events thus start with activated DCs producing cytokines and chemokines which attract additional DCs and activate T cells. Especially T helper (Th)-1 and Th-17 cells that produce IFN-γ and IL-17, respectively, seem to be involved in the perpetuation of the inflammatory response in GCA. These cells and their cytokines contribute to both local and systemic inflammation by instigating pleotropic effects on various immune cells. IFN-γ induces the production of chemokines by vascular smooth muscle cells (VSMCs). Monocytes and macrophages migrate to the vessel wall in response to these chemokines. In addition, IFN-γ activates macrophages, which are crucial in the destruction and remodeling of the vessel wall and may fuse to form multinucleated giant cells, which is one of the pathological hallmarks of GCA. IL-17 has pro-inflammatory effects on macrophages, neutrophils, endothelial cells and fibroblasts (61).

Following the activation of the adaptive arm of the immune system, an amplification of the local immune response takes place, causing vascular wall remodeling and disruption including neoangiogenesis fueling the inflammation and facilitating further immune cell recruitment to the vascular wall. Vessel-infiltrated macrophages appear to be primarily responsible for these effects, since they produce chemokines, damaging reactive oxygen species (ROS) and matrix metalloproteinases. Furthermore, macrophages produce pro-inflammatory cytokines such as TNF-α, IL-1β, IL-7 and IL-33 of which local production may translate into systemic effects. Giant cells and macrophages produce platelet-derived growth factor (PDGF) and vascular endothelial growth factor (VEGF) which can activate VSMCs (62–64). Activated VSMCS differentiate into myofibroblasts after migrating to the intima of the vascular wall. This results in intimal hyperplasia and luminal occlusion (62). The different phases in the pathogenesis of GCA have been nicely illustrated previously in a review paper by Samson et al. (61).

In the 1980’s and 90’s many studies were directed toward elucidating if circulating CD8+ T cells could act as a possible biomarker for GCA disease activity. However, during that period many studies did not differentiate between GCA and polymyalgia rheumatica (PMR), a rheumatic disease that often overlaps with GCA. Nevertheless, some studies found that CD8+ T cells were decreased in these patients (65–67), but it is highly likely that these studies were confounded by glucocorticoid treatment (68–72). It was not until recently that the possible role of CD8+ T cells gained more interest (34), as CD8+ T cells were detected at the site of inflammation in GCA-affected lesions. To which extent CD8+ T cells contribute to disease pathogenesis will be addressed in this review (**Supplementary Table 1**).

## Phenotype of CD8+ T Cells in Circulation and Affected Tissues of GCA Patients

As described previously, infectious triggers have been associated with the onset of GCA pathogenesis, but endogenous triggers could be involved as well. To assess whether a specific (viral or self) antigen causes activation and differentiation of CD8+ T cells in GCA, several studies assessed the clonality and TCR repertoire of these cells. Whereas one study found that circulating CD8+ T cells were clonally expanded in GCA patients (34), other studies found no differences in clonal expansions between HCs and GCA/PMR patients (73, 74). Clearly, these data are not conclusive on the involvement of cognate antigens in the development of GCA. However, one of the latter studies reported that even though no differences in clonal expansions were found between patients and controls, the TCR repertoire with regard to Jβ gene segments itself seemed to differ (74). In addition to this, another study found that the expression of TCR Vα and Vβ genes were different between T cells in peripheral blood and vascular tissues. The authors state that this difference may be due to local expansion of T cells caused by either recruitment of specific T cells or local proliferation of these T cells. In this study, no distinction could be made between TCR genes of CD4+ and CD8+ T cells in vascular tissues (75). Most importantly, patients and controls in these studies investigating TCR diversity were not controlled for CMV status or matched for HLA polymorphisms, factors associated with TCR diversity (76). Thus, it remains to be identified whether CD8+ T cells are clonally expanded by a response toward shared self or foreign antigens, such as derived from infectious agents. Knowledge on CD8+ T-cell clonality and TCR repertoire can aid our understanding of selective CD8+ T-cell expansion during the early phases of the disease or may inform on risk factors for development of the disease.

In a recent study, Samson and co-workers performed a detailed analysis of the phenotype of circulating CD8+ T cells in GCA (34). In this study, higher percentages of circulating cytotoxic CD8+ T cells, defined by perforin and granzyme B expression, and higher systemic levels of soluble granzyme A and B were observed. Furthermore, Tc-17 cell frequencies were found increased in GCA patients as well. Since GCA patients had higher frequencies of CXCR3+ CD8+ T cells and higher systemic levels of the ligands CXCL9, -10 and -11, Samson et al. hypothesized that CXCR3-expressing CD8+ T cells migrate to the tissue in response to those chemokines, and subsequently become activated by an unknown trigger.

Several studies identified CD8+ T cells at the site of tissue inflammation in GCA by immunohistochemistry on temporal artery biopsies (TAB). TAB tissues are often taken for diagnostic purposes, and provide an important source of information to unravel the disease pathogenesis of GCA. Three vessel wall layers can be distinguished in a TAB; from outside in: the adventitia, the media and the intima. CD8+ T cells are present in TABs, especially in the adventitia and media layers, but are less abundant than CD4+ T cells (69). Schaufelberger et al. reported that CD8+ T cells comprised 12%-46% of total T cells in TABs (75).

As mentioned above, Samson et al. hypothesized that CXCR3-expressing CD8+ T cells migrate to the tissue in response to CXCL9, -10 and -11. CXCR3+ CD8+ T cells were indeed found in TAB tissue, as well as the CXCR3 ligands CXCL9 and CXCL10. These findings led the authors to propose an adaptation of the hypothetical pathogenic model of GCA, one where CD8+ T cells have a role in aggravating the local immune response. IFN-γ produced by Th1 cells can trigger the release of, among others, CXCL9, -10 and -11 by VSMCs. This in turn can lead to recruitment of CXCR3-expressing cells, such as CXCR3+CD8+ T cells. After activation by an unknown trigger, CD8+ T cells start to produce cytokines such as IL-17 and IFN-γ. IFN-γ production by CD8+ T cells triggers additional release of chemokines, creating a feed-forward loop of additional recruited and activated CD4+ and CD8+ T cells (34). Interestingly, in this study it was also found that strong CD8+ T-cell infiltration in TABs was associated with a more severe disease course and associated with more visual disturbances. This finding, however, has not yet been confirmed by other studies in an independent cohort of patients.

Taken together, although it remains to be elucidated which factors activate CD8+ T cells in the vascular wall, it is clear that CD8+ T cells are present in GCA inflamed tissue. Studies on chemokine expression suggest involvement of the CXCL9/10/11-CXCR3 axis. Although granzyme B and perforin- expressing CD8+ T cells are elevated in blood of GCA patients, it remains to be elucidated whether these cells contribute to vasculitis development.

## Effect of Age on CD8+ T-Cell Phenotype in Circulation and Affected Tissues of GCA Patients

Several studies looked into the aging-related phenotype of CD8+ T cells in GCA. Aging is strongly associated with an increase of CD8+CD28- and to a lesser extent also CD4+CD28- (41, 77). In addition, T cells lacking the co-stimulatory receptor CD28, often upregulate other co-stimulatory receptors, such as natural killer group 2 member D (NKG2D). Studies on CD8+CD28- frequencies in GCA are inconsistent. Whereas one study described similar absolute and relative values of CD8+CD28- cells in GCA/PMR patients compared to HCs (73), another study reported higher frequencies of CD3+CD8+CD28- cells in GCA/PMR patients (78). However, the first study included treatment-naive patients only whereas the second study reported on patients of whom almost all were on glucocorticoid treatment. Furthermore, both studies did not report the CMV status of their study cohort, even though CMV status is well-known to increase the numbers and percentages of CD8+CD28- significantly. Also the finding that the frequency of circulating NKG2D-expressing CD8+CD28+ T cells was higher in GCA/PMR patients than HCs (78) could be confounded by glucocorticoid treatment. Indeed, the authors also report that patients on high-dose glucocorticoid treatment had higher NKG2D expression on CD8+CD28+ T cells than patients on low-dose glucocorticoids.

In TAB tissues of GCA patients, both NKG2D-expressing T cells and expression of one of its ligands MHC class I polypeptide-related sequence A (MICA) were detected. MICA was present on endothelial cells in the intima and on endothelial cells surrounding the vasa vasorum. Furthermore, other cells in the intima and adventitia showed moderate MICA expression whereas VSMCs in the media showed strong expression. Lymphocytes and giant cells were MICA positive as well. Although the authors did not formally proof expression of NKG2D by CD4+ or CD8+ T cells in TABs, staining of consecutive sections suggested that NKG2D was predominantly expressed by CD4+CD28- T cells, because the majority of T cells in TABS were CD4+ and CD28- (78). However, double-staining of NKG2D and CD8 and/or NKG2D and CD3/CD4 should be performed to confirm this finding, as NKG2D is generally mostly expressed by CD8+ T cells and NK cells.

Together, these findings do not support the contention that CD8+ T cells of GCA patients have a more age-associated phenotype than HCs, for instance by upregulation of NKG2D to compensate for downregulation of CD28. Even though NKG2D-expressing T cells were found in the vascular wall, we cannot yet conclude whether these cells are CD4+ or CD8+. However, the finding that MICA was expressed throughout the whole tissue suggests that MICA could be one of the ligands that activates NKG2D+CD8+ T cells in GCA lesions. Importantly, as before, none of the studies described in this section controlled for CMV serostatus, which is crucial when investigating age-associated CD8+ phenotypes.

## Failing Regulation: Regulatory CD8+ T Cells in GCA and Aging

Studies focusing on the functionality of CD8+ T cells in GCA are scarce. The only functional data available on CD8+ T cells is of a particular subset of CD8+ T cells, the CD8+ Tregs. Sufficient regulatory function of T cells is necessary to prevent the activation of autoreactive T cells. It has been postulated that CD8+ Tregs are essential in peripheral tissues such as secondary lymphoid organs (79). Circulating CD8+ FoxP3+ Tregs indeed co-express CCR7+, involved in lymphocyte homing to secondary lymphoid organs. CD8+ Tregs are thus present in secondary lymphoid organs, where they produce NADPH oxidase 2 (NOX2) in vesicles to suppress activation and expansion of CD4+ T cells. In aged individuals, the CD8+ Tregs demonstrated NOX2 deficiency which may underly the failing suppression of the immune response. This effect was even more pronounced in GCA patients (80). This was taken to suggest that CD8+ Tregs are important in the starting phase of the disease, because dysfunctional CD8+ Treg could cause unopposed CD4+ T-cell priming in secondary lymphoid organs. Unopposed T-cell priming could result in excessive inflammatory T-cell responses. Indeed, frequencies of CD8+CCR7+ Tregs expressing NOX2 were lowered in GCA, independent of glucocorticoid use, when compared to age matched controls (6% vs 23%). Interestingly, frequencies of NOX2+CD8 Tregs did not differ between patients with small-vessel vasculitis and age-matched controls: 46% of their CD8+CCR7+ Tregs expressed NOX2, against 40-50% in young healthy donors (80). Notably, the small vessel vasculitis patients had no antibodies against PR3 and MPO and were on glucocorticoid treatment.

Thus, it cannot be excluded that glucocorticoid treatment may have preserved CD8+CCR7+NOX2+ Treg frequencies. The suggestion that frequencies of CD8+ Tregs differ between different forms of vasculitis are interesting but, as this notion is based on a single report, these findings require independent confirmation.

In a follow-up study the molecular mechanisms underlying the aberrant function of CD8+ Tregs in GCA were studied. Here the authors used a mouse model in which vasculitis was induced in engrafted human arteries. Transfer of CD8+ Tregs from HCs prevented CD4+ T-cell expansion in the spleens of these mice and inhibited vessel wall invasion of CD3+ T cells. In contrast, transfer of CD8+ Tregs from GCA patients had no beneficial effects. This was caused by aberrant signaling through the NOTCH4 receptor leading to dysfunctional CD8+ Tregs in GCA (81).

Together these studies suggest a role for this rare CD8+ Treg subset in prevention of disease onset in GCA patients. Although this is an interesting notion it would require further substantiation and may await technological advances as the frequency of CD8+CCR7+ Tregs is very low even in HC.

## Function of CD8+ T Cells in GCA: Insights From Transcriptome Studies

In GCA, a transcriptome study has been performed on CD4+ and CD8+ T cells of 16 GCA patients with the aim to identify gene expression profiles that could aid in confirming diagnosis and in defining predictive biomarkers. In this study, transcription profiles of CD4+ and CD8+ T cells were assessed at six timepoints in GCA patients, from acute phase to 12 months, and at two time points in HCs. In the acute phase (T1), 288 genes were differentially expressed by CD8+ T cells and 196 by CD4+ T cells compared to HCs. The authors hypothesized that gene expression profiles would normalize after 12 months, and that genes that remain differentially expressed compared to HCs may be of clinical interest. In CD8+ T cells, two genes were differentially expressed at 12 months compared to HCs. These genes were *SGTB* which is associated with neuronal apoptosis and *FCGR3A* which is associated with susceptibility to another large vessel vasculitis: Takayasu arteritis. The implications of these differentially expressed genes for the pathogenesis of GCA are still unclear. However, the authors also correlated gene expression to disease symptoms and found that *IL32* was associated with a history of PMR, visual disturbance and raised neutrophils in the acute phase, and bilateral blindness and death within 12 months (82).

## Pathogenesis of GPA

GPA is also an aging-associated form of vasculitis as the typical age of onset is around 45 to 65 years of age. Notably, GPA not only affects adults, but also children – albeit rarer than adults. GPA affects especially the small- to medium-sized vessels. In GPA, the onset of the disease is most likely the result of a complex interplay between genetic background and environmental factors (4, 83, 84). In PR3-AAV, genome-wide association studies have revealed an association with HLA class II genes, in particular with HLA-DPB1*04:01 (85, 86).

Before the onset of symptoms, central and peripheral T and B cell tolerance toward PR3 is lost which leads to the generation of autoreactive T and B cells and results in the production of PR3-ANCAs that are characteristic for this disease. Although there is some evidence that defective Treg function and lower numbers of Bregs may contribute to loss of tolerance toward PR3 in GPA, the immunopathogenesis of the disease is far from understood.

More knowledge exists on the effector phase of the disease in which ANCA-mediated activation of primed neutrophils causing blood vessel injury is considered to be a central event (4, 87).

One of the most severe disease manifestations of GPA is the development of necrotizing crescentic glomerulonephritis (NCGN). Besides GPA, NCGN is also a frequent manifestation in microscopic polyangiitis (MPA), another form of ANCA-associated vasculitis (AAV) characterized by an autoimmune response against myeloperoxidase (MPO). Here, we will mainly focus on GPA, but studies often include both GPA and MPA patients, especially those focusing on renal disease manifestations. NCGN is characterized by necrosis of the glomerular capillary loops, after which fibrin, red blood cells, lymphocytes and macrophages seep into the urinary space surrounded by Bowman’s capsule. Subsequently, parietal epithelial cells start to proliferate which leads to glomerular crescent formation. Due to excessive inflammation, Bowman’s capsule is destructed, and glomerulosclerosis may develop leading to renal function loss. Moreover, interstitial infiltrates surrounding the necrotic lesions of glomeruli as well as inflammation of small arteries in the tubulointerstitium are commonly observed as well (4).

Studies on renal biopsies of GPA patients have demonstrated the presence of CD8+ T cells in periglomerular areas, the majority of which were located adjacent to Bowman’s capsule (88). Similarly, CD8+ T-cell infiltration has been documented in renal tissues of untreated MPO-ANCA positive MPA patients as part of the inflammatory infiltrate in the interstitium. Interestingly, in these studies interstitial CD8+ T-cell numbers, as well as those of CD4+ T cells and macrophages correlated inversely with renal function (89) an observation that has been corroborated by others (90). Collectively, these observations suggest that CD8 T cells are active contributors to disease pathogenesis in GPA. In the next sections, we will review the current knowledge on CD8+ T-cell phenotypes and function in GPA and discuss how these may link to disease development and progression (**Supplementary Table 2**).

## Phenotype of CD8+ T Cells in Circulation and Affected Tissues of GPA Patients

In GPA several studies have interrogated the phenotype of CD8+ T cells in the circulation and in affected organs such as the kidneys and lungs. The frequencies of circulating CD8+ T-cell differentiation subsets did not differ between GPA patients and HCs (91). In lung biopsies of untreated newly-diagnosed GPA patients, CD4+CD45RO+ and to a lesser extent CD8+CD45RO+ cells were found (33). Interestingly, in renal biopsies it has been reported that two-thirds of the total T-cell infiltrates is comprised of CD8+ T cells, suggesting differences in infiltrating CD4/CD8 ratios between affected tissues in GPA (88).

To assess whether CD8+ T cells are active contributors to disease pathogenesis, cytokine production and expression of activation markers as well as mechanisms of cell migration have been studied. Circulating CD8+ T cells in GPA patients were found to produce more IFN-γ compared to those from HCs (92). Moreover, in lung tissues of GPA patients increased IFN-γ gene expression has been reported compared to disease control tissue although in this study it was not established whether CD4+ or CD8+ T cells are the main producers of IFN-γ in GPA-affected tissues (33).

In 2008, Iking-Konert and colleagues provided evidence for the activation of CD8+ T cells during active disease indicated by the presence of CD11b-expressing CD8+ T cells in GPA and MPA patients. CD11b was exclusively expressed by CD8+CD28- cells in patients in remission, whereas in active disease a population of CD11b+CD28+ within the CD8+ T cell population appeared that was less prevalent in healthy donors (mean 8.9% versus 1.2% in HCs). Expression of CD11b, the α-chain of the β2 integrin Mac-1, is upregulated upon activation of T cells. Yet, whereas CD11b expression persisted on activated T cells, these cells were found to show progressive loss of CD28 expression. Therefore, the authors concluded that the CD8+CD28+CD11b+ cells must be a transient phenotype of activated T cells (93). However, as absolute numbers were not reported, it cannot be concluded that there is an actual shift of CD11b+CD28+CD8+ toward CD11b+CD28-CD8+ T cells in GPA/MPA patients.

Besides activation markers, additional studies have demonstrated that circulating CD8+CD45RO+ T cells in GPA display increased expression levels of the chemokine receptors CCR3 and CCR5 on CD8+CD45RO+ cells suggesting their readiness to respond to chemotactic gradients (94).

Furthermore, the expression of XCL1, a chemokine specifically targeting lymphocytes, was found to be increased in circulating CD4+ and CD8+ T cells in GPA (95) as well as in the renal interstitium of affected kidneys. In these renal tissues, XCL1 was co-expressed by CD4+ and CD8+ T cells (95). As XCL1 is a strong attractor for T cells, XCL1 expression might induce more interstitial T-cell infiltration.

In short, in GPA circulating CD8+ T cells appear to have an activated phenotype defined by CD11b expression, but it remains unclear if these circulating cells infiltrate the tissues despite the fact that CD8+ T cells can readily be detected in renal and lung biopsies. Further studies should investigate whether CD8+ or CD4+ T cells are the major producers of IFN-γ in GPA affected tissues, as this could aid in unraveling their contribution to disease pathogenesis. In addition, XCL1 expression could act as an amplifier of CD4+ and CD8+ T-cell migration to the renal tissues.

## Effect of Age on CD8+ T-Cell Phenotype in Circulation and Affected Tissues of GPA Patients

Given that GPA is predominantly a disease of the elderly, there has been an increased interest in studying the impact of immune aging on disease pathogenesis. As described previously, immune aging is associated with a decrease in CD28 expression by CD8+ T cells especially. A number of studies have now confirmed increased frequencies of CD28- T cells in GPA, particularly within the CD8+ T-cell compartment (96–99).

Loss of CD28 expression is associated with a poor response to TCR stimulation, and has therefore been associated with senescence. Consequently, the telomere length of circulating T cells in GPA patients has been assessed as well. T cells of GPA patients demonstrated indeed shorter telomere lengths than age-matched HCs. However, lack of CD28 and shorter telomere length was especially observed in GPA patients with long lasting disease suggesting recurring activation of the same T cells. Also, since these studies were performed on total T cells, it remains unclear whether shorter telomere lengths are characteristic of either CD4+ and CD8+ T cells lacking CD28, or both (96). Compared to disease controls, increased proportions of CD28 negative cells have also been detected in bronchoalveolar lavage (BAL) fluid and in biopsies from the upper respiratory tract of GPA patients. Again, however, it is unclear whether the CD28- cells in these biopsies were CD4+ or CD8+ T cells (99).

As previously described, the co-stimulatory receptor NKG2D has been implicated in the disease pathogenesis of several forms of vasculitis including KD, TA and GCA. As CD8+ T cells lacking CD28 often upregulate NK-like co-stimulatory receptors, NKG2D and its ligand MICA have been considered important markers in age-associated vasculitides such as GPA. In one study investigating kidney biopsies of active untreated GPA patients, MICA expression was detected on peritubular and glomerular capillaries as well as on epithelial cells. In this study, CD8+ T cells and NKG2D-expressing cells were also found around tubular and glomerular capillaries although it was not determined whether the NKG2D-expressing cells were also CD8+ (100).

Late-stage differentiated cells such as CD8+ T_EMRA_ cells often express CD57. CD57 expression is increased upon aging and CD57+ cells can produce pro-inflammatory cytokines. In GPA and MPA patients younger than 40 years of age, the frequency of circulating CD8+CD57+ cells was found increased compared to age-matched healthy donors. Increases in CD8+CD57+ cells were associated with severe disease and multiple organ involvement (101). However, another study found no differences in percentages of CD28- and CD57+ cells in CD4+ and CD8+ T cells in GPA and MPA patients versus HCs (102).

When interpreting data on phenotypes of immune cells in general, and CD8+ T cells in particular, it is important to take CMV serostatus into account. Importantly, the studies described above did not correct for CMV serostatus, even though CMV infections generally lead to increased numbers and frequencies of CD4+CD28- and CD8+CD28- T cells and late stage differentiated cells (42, 47, 103, 104). Indeed, also in GPA, CMV serostatus has been associated with high frequencies of CD28- T cells and CD57+ T cells (102). Regarding CD28 expression, concomitant infections with CMV and EBV, as determined by the presence of antigen-specific memory T cells, have been associated with a loss of CD28 expression by circulating CD8+ and CD4+ T cells in GPA patients. Interestingly, cellular positivity for CMV or EBV only was not associated with this phenotype in GPA patients, nor was CMV and EBV negativity. However, no differences in frequencies of CMV or EBV antigen-specific cells were found within the total CD8+ T-cell and CD8+CD28- population in GPA patients and HCs. This suggests that CMV and EBV infections exert indirect effects on CD8+ T cells which causes the expansion of CD8+CD28- cells in GPA patients, such as through bystander activation and/or cytokine mediated expansion (97). Expansion of non-antigen-specific cells by inflammatory processes are especially pronounced during later stages of disease or infection, whereas initial immune responses are caused by antigen-specific cells (105).

Another study reported on lower CD28 expression on CMV-specific CD8+ T cells in GPA patients than in HCs (106). The CMV-specific CD8+ T cells were either CD28-CD27+ or late stage memory cells defined by loss of CD28-CD27- expression. However, frequencies of CD28- cells were also lower in the non CMV-specific CD8+ T-cell repertoire of GPA patients. The authors suggested that higher CD28- frequencies in GPA patients could be a result of the disease itself rather than CMV status. However, as mentioned before, CMV and EBV infection could have an indirect effect on the expansion of CD8+CD28- cells in GPA as well.

In summary, it remains unclear whether markers of aging are more frequently expressed in GPA patients, as CMV and EBV status may modulate the expression of these markers, confounding data interpretation and comparison, especially with regard to CD28 expression. Also, methodological differences between studies such as the use of fresh whole blood versus freshly isolated or cryopreserved PBMCs may have influenced the reported CD28 expression data (107). Since NKG2D has been implicated in several forms of vasculitis, additional studies into the spatial temporal expression and functional role of this receptor in the inflammatory response in GPA are warranted.

## Function of CD8+ T Cells in GPA: Insights From *In Vitro* Studies

To better understand if and how CD8+ T cells contribute to GPA pathogenesis, studies investigating their functionality are imperative. However, data on the function of CD8+ T cells in GPA is limited. As mentioned earlier, CD8+CD28+CD11b+ cells were found to be increased in the circulation of active GPA patients. These cells are also capable of producing IFN-γ *in vitro*. Co-cultures with polymorphonuclear neutrophils (PMN) showed that IFN-γ-producing CD8+C11b+ T cells can activate PMN from GPA patients to express MHC class II (93). Previously, it was found that PMN of GPA patients acquire characteristics of antigen presenting cells by expressing MHC class II, a phenotype not present in HCs (108). As described earlier as well, circulating CD8+ T cells expressing the chemokine XCL1 were elevated in GPA patients and XCL1 was also expressed in the renal interstitium. *In vitro* stimulation of PMN with XCL1 led to increased production of the pro-inflammatory cytokine IL-8 (95). Collectively, these studies imply that CD8+CD28+CD11b+ and CD8+CXCL1+ cells could exert pro-inflammatory effects on PMN. However, to which extent these processes contribute to the disease pathogenesis in GPA is currently unknown and requires further study.

## Function of CD8+ T Cells in GPA: Insights From Transcriptome Studies

Two transcriptome studies from the same group provide evidence that CD8+ T cells could play a role in GPA/AAV based on the transcriptional profile of these cells during active disease. These studies were originally designed to understand the molecular basis of the considerable variation that exists between autoimmune patients regarding clinical course and outcome of their disease. Furthermore, these transcriptome studies aimed to discover biomarkers that could aid in developing strategies for personalized medicine. In the first study, microarray analysis of purified total CD8+ T cells from patients with active disease revealed that two distinct CD8+ expression signatures may serve to predict the clinical course of AAV and other autoimmune diseases such as SLE. Patients with a relapsing course of their diseases and a poor prognosis were found to have a CD8 transcriptional profile enriched in genes involved in the IL-7R and TCR pathway and effector memory cells. Further analysis showed that these patients also had an expanded memory CD8+ T-cell population. Based on these results, the authors postulated that via enhanced IL-7R and TCR signaling, CD8+ T-cell proliferation in response to antigens increases leading to an expanded memory population. In autoimmune diseases, autoreactive cells with enhanced IL-7R and TCR signaling pathways expand more easily upon restimulation, which leads to more effector cells thereby promoting tissue damage (109). In a subsequent study, McKinney and colleagues demonstrated that a transcriptome profile of CD8+ T cells resembling an exhausted signature correlates with good outcome in autoimmune diseases such as AAV (110). Based on these results, the authors suggested that targeted induction of T-cell exhaustion could be a novel treatment strategy for autoimmune diseases.

## Function of CD8+ T Cells in GPA: Insights From Animal Models

Unlike GCA, animal models have been developed for MPO-ANCA-associated vasculitis that have been instrumental in dissecting the various effector mechanisms involved in disease development. In most of these models the kidney is the main organ affected mimicking human focal necrotizing crescentic glomerulonephritis (FNGN).

In one such model, autoimmunity to MPO is induced in mice by immunization with human MPO which results in a humoral (MPO-ANCA) as well as a cellular immune response to autologous mouse MPO. In this model, an additional challenge with heterologous anti-mouse glomerular basement membrane antibodies recruits neutrophils to glomeruli causing local deposition of MPO in glomerular capillaries where it can be recognized by effector T cells triggering glomerulonephritis (GN) development. Initial studies in this model demonstrated an important role for CD4+ effector T cells as CD4+ T-cell depletion in the effector phase markedly attenuated GN development (111). More recently, this model has been employed to study the role of CD8+ T cells in the development of tissue injury as well. Systemic depletion of CD8+ T cells in the effector phase reduced GN development accompanied by diminished renal production of IFN-γ and TNF-α and less glomerular macrophages. In the same study, the authors generated MPO-specific CD8+ T-cells clones which upon transfer mediated glomerular injury when MPO was deposited in glomerular capillaries (112). Collectively, these studies support a pathogenic role for antigen-specific CD8+ T cells in AAV pathogenesis

In an attempt to assess the role of CD8+ T cells in glomerular crescent formation more directly, Chen and colleagues recently generated mice that express the model antigen EGFP on podocytes. Upon transfer of EGFP-specific CD8+ T cells these mice developed crescentic glomerulonephritis but only when injury to the glomerular filtration barrier was induced concomitantly by injection of a nephrotoxic serum. These observations imply that the nephrotoxic serum disrupts the physical barrier that would otherwise prevent recognition of EGFP by CD8+ T cells. From these data a model emerges in which antigen-specific CD8+ T cells can infiltrate the urinary space through Bowman’s capsule when it is breached. In turn, these CD8+ T cells may interact with podocytes bearing their cognate antigen, which accelerates kidney injury and further stimulates the formation of crescents (113).

However, whether these observations can be translated to the situation in humans is as yet unclear. Theoretically, podocytes could present ANCA antigens as at least MPO has been detected in and around podocytes in renal biopsies of AAV patients (89). Moreover, focal endocapillary inflammation is commonly observed in early lesions in GPA as well. This suggests that an initial inflammatory response in the glomerular capillaries may lead to leakage of ANCA antigen-specific CD8+ T cells through Bowman’s capsule where they may interact with the PR3 or MPO bearing podocytes and accelerate crescent formation (114).

## Discussion

### Are CD8+ T Cells Active Contributors to Disease Pathogenesis or Just Bystanders?

**Figure 3:**
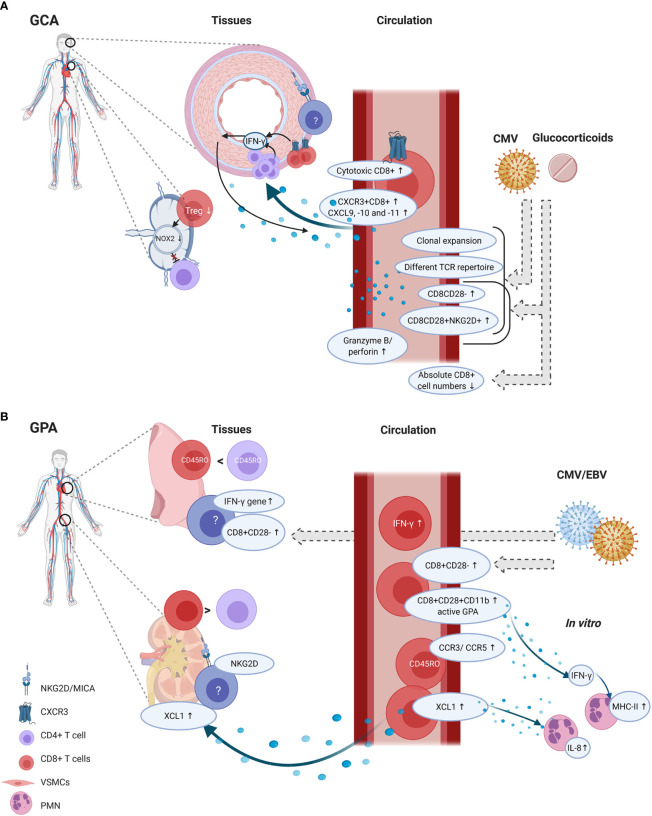
CD8+ T-cell functions and phenotype in tissue and circulation of GCA and GPA patients. GCA **(A)** In disease onset, CD8+ Tregs residing in lymph nodes could be involved, as their function decreases which could lead to unopposed CD4+ T-cell activation. In the circulation of GCA patients several phenotypic changes are present in CD8+ T cells. The percentages of cytotoxic T cells are increased alongside elevated serum levels of granzyme B and perforin. In vascular tissues, the hypothesis is that first CD4+ T cells produce IFN-γ. IFN-γ production leads to CXCL9, -10 and -11 production by vascular smooth muscle cells (VSMCs). This attracts CXCR3+CD8+ T cells which contribute to the feed forward loop by producing IFN-γ. Some studies reported on age-associated changes in CD8+ T cells, such as increased clonal expansions, a different T-cell receptor repertoire (TCR), increased CD8CD28- and CD8CD28+NKG2D+ frequencies and decreased absolute numbers CD8+ T cells. But these findings could be confounded by glucocorticoid use and/or CMV infection. GPA **(B)** CD8+ T cells are present in lung biopsies but their frequency with regard to CD4+ T cells is higher in renal tissue. In renal tissues, NKG2D expression has been found but it is unclear which cells express this marker. Furthermore, MICA expression was found. CD8+ T cells could migrate to the tissues by XCL1, and XCL1 also induces IL-8 production by polymorphonuclear neutrophils (PMN). In the circulation, also CCR3 and CCR5 was upregulated on memory CD8+ T cells, but it remains unclear how this relates to tissue migration. CD8+ T cells in the circulation show an activated phenotype as CD11b was elevated, and these cells are able to produce IFN-γ. IFN-γ levels were also elevated in the circulation and the IFN-γ gene was elevated in lung biopsies. From in vitro studies it is known that IFN-γ can induce PMN to express MHC-II molecules. Although CD8+CD28- frequencies seem increased in tissues and circulation of GPA patients, it is likely that these findings are confounded by CMV and/or EBV infections. Created with BioRender.com.

In GCA, several findings suggest that CD8+ T cells are active contributors to disease pathogenesis (**Figure 3A**). First and foremost, CD8+ T cells are clearly present in TAB tissues and one study suggested that strong CD8+ T-cell infiltration in TABs might be associated with a more severe disease course and also with more visual disturbances. Secondly, *in vitro* data showed the importance of CD8+ Tregs in inhibiting CD4+ T-cell activation. As GCA patients demonstrated impaired functioning of CD8+ Tregs, these cells could render aged adults more susceptible to disease development. Lastly, possible migratory mechanisms involving CXCR3+CD8+ T cells have been proposed. However, none of these findings conclusively confirm a pathogenic role of CD8+ T cells in GCA pathogenesis.

CD8+ T cells appear to be active contributors to GPA pathogenesis, especially in the development of glomerulonephritis (**Figure 3B**). Several observations support this contention. Firstly, CD8+ T cells are present in renal biopsies, adjacent to Bowman’s capsule. Secondly, CD8+ T cells seem to outnumber CD4+ T cells in renal biopsies. Thirdly, CD8+ T cells correlate with decreased renal function. Circulating CD8+ T cells bear a more activated phenotype and possible mechanisms involving direct effects on PMNs have been described. Finally, data from mouse models suggest that CD8+ T cells can interact with antigen-bearing podocytes in glomeruli, which accelerates kidney injury. However, the evidence that immune cells can gain access through Bowman’s capsule is still correlative, as it was only found that CD8+ T cells were more commonly present within the glomeruli when Bowman’s capsule was ruptured (113). No direct proof exists that CD8+ T cells breach Bowman’s capsule. Furthermore, it remains uncertain whether podocytes cross-present neo-epitopes or circulating antigens to CD8+ T cells (115).

Both GCA and GPA susceptibility have been associated with HLA class II genes. In GCA, associations with HLA-B, a class I molecule, have been reported as well. However, associations with HLA class I genes in GCA do not appear to be as strong as in TA. How HLA class I and/or II genes contribute to disease susceptibility in GCA and GPA is still unclear. At least in GPA, associations with HLA class II genes could also just reflect the role of the HLA molecules in peptide presentation, such as PR3 (4).

### To What Extent Does Aging Affect the Function and Phenotype of CD8+ T Cells in GCA and GPA?

Although several studies have reported that typical aging-associated phenotypes of CD8+ T cells are more frequent in GCA and GPA patients than in controls, it cannot be excluded that data have been confounded by CMV and/or EBV infection, glucocorticoid treatment and methodological differences (**Figure 3**). Also, data on clonality and TCR diversity in GCA are likely confounded by CMV serostatus and/or HLA polymorphisms. In GPA, the effect of treatment on phenotype and function of CD8+ T cells is always difficult to assess, as most studies looked into a heterogeneous group of MPA and GPA patients with large age-ranges and in different disease states. Whereas in most studies in GCA the patients were newly diagnosed and not yet on treatment, in most GPA studies patients received immunomodulatory therapy. These limitations make it difficult to compare studies and draw firm conclusions.

In this review, we discussed NKG2D as a possible marker of aging, as T cells that downregulate CD28 expression often upregulate other co-stimulatory NK markers. However, NKG2D is not an exclusive marker of aging as it is also involved in other forms of vasculitis that affect younger adults and children, such as KD and TA. Although it is still unclear to what extent NKG2D and its ligand MICA contribute to disease pathogenesis in GCA and GPA, it is interesting that NKG2D and MICA have been implicated in these other forms of vasculitis. This underlines the possible importance of NKG2D and MICA and the vascular inflammatory environment and suggests that binding of CD8+ T cells to MICA by virtue of their NKG2D receptor activates these cells to become proinflammatory.

### Future Outlook

To truly understand the role of CD8+ T cells in the pathogenesis of primary vasculitides, more integrated and in-depth analyses of CD8+ T cells in both GCA and GPA are required. As in both GCA and GPA several transcriptome studies have been performed, a comprehensive analysis of all differentially expressed genes could open up novel avenues for research on shared or distinct disease mechanisms and the exploration of new targets for disease monitoring and therapy. As an example, longitudinal profiling of GCA patients, revealed differential expression of the IL-32 gene in CD8+ T cells whereas elevated serum levels of IL-32 that correlated with disease activity have been reported in AAV (116). Thus, IL-32 could perhaps constitute an interesting lead for further study in relation to disease severity in various vasculitides. In GPA, an exhaustion profile of CD8+ T cells has been associated with favorable clinical outcome. More research into this exhausted profile would teach us more about the involvement of CD8+ T cells in disease pathogenesis and potentially uncover novel targets for therapy.

Although the standard therapies for GCA and GPA are not designed to directly target CD8+ T cells, studying the effects of these treatments on CD8+ T cell function could aid in unraveling their role in these diseases. As an example, one study in AAV found that whereas rituximab treatment did not affect CD4+ and Treg frequencies, it was associated with reduced CD8+ T_EMRA_ frequencies and circulating chemokine and cytokine levels. Interestingly, co-cultures of CD8+ T cells and autologous B cells from AAV patients resulted in enhanced production of proinflammatory cytokines indicating a pathogenic crosstalk between B cells and CD8+ T cells (117).

Studies on CD8+ Tregs in GCA should be confirmed by other research groups possibly aided by advanced technologies such as single cell sequencing. If confirmed, revival of these non-functional CD8+ Tregs would certainly be of interest in prevention of aging-associated pathologies such as GCA.

In both GCA and GPA, more detailed interrogation of vasculitic tissues is required ideally employing state of the art technologies such as imaging mass cytometry. Single cell RNA sequencing of TAB tissue digests from microdissected lymphocyte infiltrates can also be performed to investigate CD8+ T-cell heterogeneity at the single cell level to obtain better insights into CD8+ T-cell function in the lesional environment. Furthermore, this would also help to investigate whether CD8+ T_RM_ cells are present and involved in disease pathogenesis.

Aberrant DNA methylation and microRNA expression in CD8+ T cells have been linked to autoimmune diseases such as multiple sclerosis, type 1 diabetes and SLE. Thus, for both GCA and GPA it would be interesting to investigate the epigenetic profile of CD8+ T cells as well, especially because emerging evidence suggests that microRNAs, histone modifications and DNA methylations can lead to dysfunctional CD8+ T cells (118).

Another area of research relevant to delineate the effects of CD8+ T cells on GCA and GPA includes the microbiome. It is well known that the microbiome has profound effects on the wellbeing of people, for instance by regulating immune homeostasis. Alterations of the microbiome, generally referred to as dysbiosis, have been linked to states of aberrant immune activation implicated in various chronic autoimmune diseases (119). Many studies on the effects of the microbiome on the immune response are focused on finding anti-tumor properties of certain bacterial strains. For instance, a recent study in a mouse model of inflammation-associated tumorigenesis found that gut microbiota can have direct effects on CD8+ T-cell responses (120). Furthermore, another study demonstrated that a combination of several bacterial strains isolated from healthy human feces promoted the development of IFN-γ-producing CD8+ T cells in mice and enhanced the efficacy of immune checkpoint blockade therapy in tumor models (121). However, these observations also suggest that dysbiosis of the gut microbiome may enhance auto-inflammatory effects, for instance by boosting IFN-γ production by CD8+ T cells. So far, data on the microbiome of the gut or other body niches in GCA and GPA is limited but this certainly warrants further investigation.

## Conclusion

Taken together, in vasculitic diseases, CD8+ T cells may be active contributors to disease pathogenesis via their effector function, likely to enhance local inflammation and tissue damage, and/or via their failing regulatory function. Both aspects deserve further exploration employing novel technologies in concerted actions involving well-described patient cohorts.

## Author Contributions

The following authors contributed to design of the article (RR, AB, PH, and EB) and all authors contributed to the content and writing and approved the submitted version. All authors contributed to the article and approved the submitted version.

## Funding

This work was in part funded by the Graduate School of Medical Sciences of Groningen.

## Conflict of Interest

AB was a consultant for Grünenthal Gmbh until 2017. EB as an employee of the UMCG received speaker/consulting fees from Roche paid to the UMCG.

The remaining authors declare that the research was conducted in the absence of any commercial or financial relationships that could be construed as a potential conflict of interest.
