# Supplementary material for: CD8+ T Cells in GCA and GPA: Bystanders or Active Contributors?
Source: Front Immunol. 2021 Mar 18;12:654109. doi: 10.3389/fimmu.2021.654109 (PMC8015776; doi:10.3389/fimmu.2021.654109)
Supplement: Supplementary file 1 [file DataSheet_1.docx]

**Supplementary table 1: Overview of articles about CD8+ T cells in GCA**

|  | **Disease, n** | **Disease duration and treatment** | **Age, years**  **sex, male/ female (M/F)** | **Methods*** | **Results** | |
| --- | --- | --- | --- | --- | --- | --- |
|  |  |  |  |  | **Circulation/ *in vitro*** | **Tissue** |
| Benlahrache (67) | PMR, 28  GCA, 7  HCs, 35 | Mean 2 years  Glucocorticoids n=24 | PMR/GCA: Mean 72.6, 7M 28F  HCs: mean 77.2,  7M 28F | Indirect IF of  frozen mononuclear cells. | Lower percentages of T cells and CD8+ T cells in patients than in HCs. | n.a.** |
| Banks (69) | Circulation:  GCA/PMR, 6  Tissue: GCA/PMR, 14 | Mean, 6 months  Circulation:  Before treatment  Tissue: Glucocorticoids n=6 | GCA: mean 73, 2M 12F | Frozen TABs  Flow cytometry on heparin WB and PBMCs. . | Circulating CD8+ T cells within normal range. | In two tissues, 25-50 % of tissue infiltrates were CD8+ T cells and in 12 tissues, 0-25% were CD8+ T cells. |
| Andersson (68) | PMR, 4  GCA, 6  HCs, 108 | Before treatment | PMR/GCA: mean 68, 4M 6F  HCs: 20-60 , 108M | Flow cytometry on heparin WB. | No differences in relative percentages of CD8+ T cells in patients compared to normal reference values. | n.a. |
| Dasgupta (65) | PMR/GCA, 33  HCs, 20 | Before treatment |  | Cell counts of WB.  Flow cytometry on fresh PBMCs. | Decrease in absolute and relative numbers/ percentages of CD8+ T cells which normalized after 24 months | n.a. |
| Macchioni (66) | PMR/GCA,22  HCs, 61 | Mean, 7 weeks  Before treatment | PMR/GCA: mean 70.2, 6M 16F | Flow cytometry on EDTA WB. | Decrease in absolute and relative numbers/ percentages of CD8+ T cells. | n.a. |
| Pountain (71) | GCA/PMR, 36  HCs and Osteoarthritis, 36 | Before treatment | GCA/PMR: Median 70 | Cell counts and flow cytometry on WB. | No differences in absolute and relative numbers/percentages of CD8+ T cells in patients and controls.  Patients with most severe disease had reduced CD8+ T cell numbers. | n.a. |
| Udhammar (70) | PMR, 23  Positive TAB, 1  HCs, 14 | Mean, 24 months  Before treatment | PMR: mean 70.7, 7M 16F  HCs: mean 73.5, 6M 8F | Cell counts and flow cytometry on EDTA WB. | No differences in absolute and relative numbers/percentages of CD8+ T cells in patients and controls. | n.a. |
| Martinez-Taboada (74) | PMR, 12  GCA, 6  HCs, 9 | Before treatment | PMR: mean 72.9, 2M 10F  GCA: mean 73.8, 2M 4F  HCs: mean 69, 4M 5F | CD8+ T cells isolated by magnetic beads of and  flow cytometry and Vβ specific PCR, spectratyping on fresh PBMCs. | PMR/GCA patients had clonally expanded CD8 populations.  Frequency of clonal expansion was not different from HCs. | n.a. |
| Martinez-Taboada (72) | PMR, 28  GCA, 6  Disease controls, 18 | Before treatment | GCA/ PMR: mean 69.9, 10M 24F Disease controls: mean 59, 3M 14F | Flow cytometry on EDTA WB. | No differences in absolute and relative numbers and percentages of CD8+ T cells in patients and controls |  |
| Lopez-Hoyos (73) | PMR, 18  GCA, 5  HCs, 23 | Before treatment | GCA/PMR: mean 73.2, 12M 11F  HCs: mean 73, 9M 14F | HLA typing on PBMCs. Flow cytometry on WB | Percentages and absolute numbers of CD8+ T cells were comparable in patients and controls. No differences in percentages and absolute numbers of CD8+CD28+, CD8+CD57+, CD8+CD45RA+, CD8+CD45RO+ andCD8+CD25+ subsets.  Clonal expansions were detected in CD8+ T cells of HCs and patients. | n.a. |
| Schaufelberger (75) | Circulation:  GCA, 7  Tissue:  GCA, 5 | Mean 9 weeks  1 day glucocorticoids n=2 | GCA: median 67, 1M 6F | IHC on TABs.  Flow cytometry on WB. | No expanded T-cell populations. | Six out of seven patients had expanded T-cell populations in TABs.  Between 12% and 46% of T cells were CD8+ in TABs. |
| Dejaco (78) | GCA, 16  PMR, 78  HCs, 64 | ***GCA: mean 24 months  Before treatment n=2  PMR: mean 6 months  Before treatment  n=15 | ***GCA: mean 74.5, 3M 11F  PMR: mean 70.1, 10M 57F | IF for NKG2D/CD3 double staining in three TABs. IF and IHC for NKG2D ligand expression. mRNA levels ligands by RT-PCR.  Cell proliferation experiments, intracellular IFN-γ and TNF-α staining by Flow cytometry in CD4+ and CD8+ T cells isolated by magnetic beads. | NKG2D was expressed by CD4+CD28- and CD8+CD28- and CD8+CD28+ cells in patients.  CD3+CD8+CD28- cells were increased in patients.  NKG2D relative expression by CD8+CD28+ cells was higher in patients.  Patients on high-dose corticosteroids had higher relative NKG2D expression by CD8+CD28+ cells. | CD3+NKG2D+ cells were found around the vaso vasorum in the adventitia.  More MICA was found in positive TABs than in negative TABs. |
| Samson (25) | GCA, 34  For spectratyping, 9  HCs, 26  For spectratyping, 7 | Before treatment  Spectratyping:  3 untreated,  6 treated | GCA: mean 75.3, 13M 21F  HCs: mean 73.1, 10M 16F | Flow cytometry on fresh PBMCs.  Luminex for serum markers. IHC on TABs.  RT-PCR, TCR Vβ spectratyping on CD8+ T cells isolated by magnetic beads. | No differences in numbers of CD8+ T cells.  Higher perturbation index in GCA patients compared to HCs illustrates presence of clonal CD8+ T cell expansion.  Increased frequency of cytotoxic CD8+ T cells. | CXCL9, -10 and -11 seems to be involved in CXCR3+CD8+ T cell migration to arterial wall.  More CD8+ T cell infiltration in TABs correlates with more severe disease. |
| Wen (80) | GCA, 13  HCs, 64 | Untreated: n=7  Glucocorticoids : n=6 | GCA: mean 72.3  HCs: range 20-84 | *In vitro* experiments performed on isolated subsets of CD4+ and CD8+ T cells from fresh PBMCs. | Deficiency of NADPH oxidase 2 accounts for CD8+ Treg failure in older donors and GCA patients. |  |
| De Smit (82) | GCA, 16  HCs, 16 | Acute phase and follow up.  Analysis took into account untreated n=6 and treated at first timepoint | GCA: mean 78.2, 2M 14F  HCs: mean 76.6, 2M 14F | CD4+ and CD8+ T cells isolated by magnet beads.  RNA isolated for RNA sequencing. | *SGTB* and *FCGR3A* remained differently expressed after 12 months. *IL32­* correlated with symptoms such as blindness. | n.a. |
| Jin (81) | GCA, 102.  Numbers vary per experiment. | Untreated: n=37 | GCA: mean 73.4, 74F | Sorted CD8+CD39+CD26- Tregs or *ex vivo* induced CD8+ Tregs from GCA patients and HCs for assessment of functionalities of CD8+ Tregs.  CD8+ Treg function was determined in NSG mice in which vasculitis was induced in engrafted human arteries. | n.a. | Functional failure of anti-inflammatory CD8+ Treg cells was a consequence of aberrant signaling through the NOTCH4 receptor. |

* TAB: temporal artery biopsy. WB: whole blood. PBMCS: peripheral blood mononuclear cells. IHC: immune histochemistry. IF: immunofluorescence. NSG: NOD scid gamma mouse

** n.a. : not applicable

***Clinical data available of 11 GCA and 67 PMR patients

**Supplementary table 2: Overview of articles about CD8+ T cells in GPA**

|  | **Disease, n** | **Disease duration, disease state and treatment** | **Age, years**  **sex, male/ female (M/F)**  **CMV** | **Methods*** | **Results** | |
| --- | --- | --- | --- | --- | --- | --- |
|  |  |  |  |  | **Circulation/ *in vitro*** | **Tissue/ Animal model** |
| Moosig (98) | GPA, 24  HCs, 17 | Mean 5.1 yrs  Active n=9  Inactive n=15  glucocorticoids n=22  cyclophosphamide n=10  methotrexate =8 | GPA: mean 56, range 36-75, 17M 7F  HCs: mean 57, range 34-75, 12M 5F | Flow cytometry on WB.  *in vitro* PBMCs stimulated with PHA and PMA.  APCs depleted with T cells incubated with IFN-γ. | Frequencies of CD4+CD28+ and CD8+CD28+ lower in GPA than HCs, after *in vitro* stimulation.  Percentages of CD28+ cells correlated negatively with disease activity. | n.a. ** |
| Lamprecht (99) | Circulation:  GPA, 10  HCs, 15  Tissue:  GPA, 10  Disease controls, 6 | Mean 3.5 yrs  Before treatment n=1  Relapse n=7  Partial remission n=2 | GPA: mean 63  Controls: mean 44  Statistically significant difference | Flow cytometry on WB and BAL fluid.  IF for T cell and CD28 double staining on biopsies. | More CD28- cells in CD8+ T cells in BAL than in blood of patients but not in controls. Total numbers of CD4+CD28- but not of CD8+CD28- was higher in BAL than blood in patients.  Total numbers of CD8+CD28- higher in patients than in HCs. | Higher fraction of CD28- T cells in CD8+ T cells in BAL of patients.  CD28- T cells found in granulomatous lesions. |
| Coulomb-L’Hermine (24) | GPA, 6  Disease controls, 6 | Untreated | GPA: range 40-73,  1M 5F | IHC on lung tissues.  In situ hybridization and PCR for cytokine expression. | n.a. | Lymphocytes in lung lesions of GPA patients are mostly memory CD4+CD45RO+ and CD8+CD45RO+. |
| Aasarød (88) | GPA, 61 | Treated: n=18 | GPA: mean 57, range 15-80 | IHC on renal biopsies. | n.a. | Intraglomerular leucocytes consisted of macrophages and T cells and no B cells. More than 2 thirds were CD8+ T cells. CD8 T cells were mainly adjacent to Bowman’s capsule |
| Ohta (92) | GPA, 7  HCs, 10 | Median 42 m.  Glucocorticoids: n=7  Cyclophosphamide: n=5 | GPA: median 49.5, range 24-68, 3M 4F | Flow cytometry on and PMA stimulation of PBMCs. | Population of Tc-1 cells in GPA was increased. | n.a. |
| Vogt (96) | GPA, 55  Disease controls, 8  HCs, 35  For telomere lengths:  GPA, 22  Disease controls, 8  HCs, 10 | None active disease  GPA > 5 yrs, n=31  GPA > 5 yrs, 1 active episode, n=13  GPA ≤5 yrs, n=11 | GPA: range 24-77 | Telomere length by southern blotting.  CD28 by cytofluorometry. | GPA > 5 yrs: short telomeres in addition to normal telomeres, indicating replicative senescence of T cell clones.  CD8+CD28+ expression lower in patients especially in GPA >5 yrs and short telomeres (n=7)  2 older donors had short telomeres | n.a. |
| Lamprecht (106) | GPA, 6  Controls, 6 | Active: n=4  Remission: n=2  All were treated | All patients and controls were CMV positive | PBMCs were incubated with HLA-restricted CMV pp65 peptide-MHC class I tetramer complexes. | HCs had lower CD28 expression on CMV-specific CD8+tet+ T-cells compared to the CD8+tet− [T-cell](https://www-sciencedirect-com.proxy-ub.rug.nl/topics/biochemistry-genetics-and-molecular-biology/t-cell)s.  CD28 was lower or absent on CMV-specific CD8+tet+ in GPA than HCs. CD28 was lower and CD27 higher on CMV-specific CD8+tet+ T-cells and CD8+tet− T-cells in patients. | n.a. |
| Lamprecht (94) | GPA, 21  HCs, 13 | Localized: n=5.  All remission  Generalized: n=16  Active: n=9  Remission: n=7 |  | Flow cytometry on PBMCs. | Fractions of CCR5+ and CCR3+ cells within the CD4+CD45RO+ and CD8+CD45RO+ memory T cell populations were significantly expanded in localized and generalized GPA. | n.a. |
| Abdulahad (91) | GPA, 57  HCs, 21 | Active: n=17,  treated n=3,  mean duration 0 m  Remission: n=40, treated n=11  Mean duration 78 m | Active: median 55, range 19-85, 10M 7F  Remission: median 57, range 17-87, 24M 16F  HCs: mean 51, range 20-83, 9M 12F | Flow cytometry on fresh PBMCs. | The distribution of naive and memory CD8+T cells did not differ between patients and HCs. | n.a. |
| Holmén (100) | GPA, 6 | Active: n=6  Without current treatment | GPA: range 36-80, 4M 3F | Kidney sections stained with anti-MICA, anti-CD8 and anti-NKG2D. | n.a. | MICA is expressed around glomerular vessels and epithelial cells. NKG2D and CD8 T cells located around tubular and glomerular capillaries. |
| Iking-Konert (93) | AAV, 90 | Active: n=10, relapse n=1, untreated GPA n=4, untreated MPA n=5  Remission: n=80  GPA n=47  MPA n=33 |  | Flow cytometry on WB.  Co-cultivation experiments with CD8+ T cells isolated by magnetic beads  and PMN | Active disease: CD8+CD28+CD11b+ cells produce IFN-γ. In HCs and patients in remission or on immunosuppressive therapy CD11b was associated with CD8+CD28- cells.  CD11b is upregulated when T cells are activated. CD11b expression persists and CD28 is lost. IFN-γ-producing T cells activate PMN to express MHC class II. | n.a. |
| Iking-Konert (101) | Exact numbers unclear  GPA, 52  MPA, 33  HCs, 34 | Active: n=5  Remission: n=80  Before treatment n=4 | Presumably:  GPA: median 36, n=9. Median 68, n=46  HCs: median 36, n=10. Median 68, n=27 | Flow cytometry on WB. | CD8+CD57+ increased with age.  In younger patients CD8+CD57+ was increased compared to HCs.  CD8+CD57+ correlated with disease severity | n.a. |
| Blaschke (95) | Circulation:  GPA, 16  HCs, 16  Tissue: GPA, 5  Disease controls, 4 | Mean 5.6 yrs  Active: n=6  Remission: n=10 | Circulation:  12M 4F  Tissue:  GPA: range 29-54 yrs. | Flow cytometry on PBMCs.  PBMCs stimulated with PMA to detect XCL1 expression.  ELISA for XCL1 levels in serum.  IHC double labelling XCL1 and CD4, CD8 and CD68.  PMN stimulated with recombinant XCL1. | XCL1 expression was higher in patients in CD4+ and CD8+ T cells.  Mainly CD8+CD28- T cells expressed XCL1.  Active patients had more XCL1+ CD4+ and CD8+.  PMNs produced IL-8 upon XCL1 stimulation.  No differences in serum XCL1 levels between all groups. | XCL1 was expressed in interstitium of renal biopsies in CD4+ and CD8+ T cells. |
| McKinney (109) | AAV, 59  SLE, 25 | Active: n=34  N=27 as validation.  Group 1: before treatment  Group 2: n=24 before treatment | Group 1: mean 57.5, 9M 10F  4 MPO, 15 PR3  Group 2: mean 62, 15M 25F  16 MPO, 22 PR3 | Transcriptional profiling of purified CD8+ T cells. | Poor prognosis is associated with IL7R pathway, TCR signaling and memory T cell transcripts, and an expanded CD8+ memory population. | n.a. |
| Eriksson (102) | GPA, 24  MPA, 10  HCs, 20 | Active: n=12 GPA, n=6 MPA  Remission: n=12 GPA, n=4 MPA | Remission: median 75, 7M 9F  Active: median 67, 12M 2F  HCs: median 70, 12F 8F | Flow cytometry on WB. | Active patients: total numbers and proportions of lymphocytes decreased. Total numbers of CD8+CD28- and CD8+CD57+ were decreased.  CMV was associated with expanded proportions of CD28- and CD57+ cells in CD4+ and CD8+ compartments. | n.a. |
| McKinney (110) | AAV, 59 |  | Group 1: mean 57.5, 9M 10F  4 MPO, 15 PR3  Group 2: mean 62, 15M 25F  16 MPO, 22 PR3 | Microarray gene expression profiling of RNA of PBMCs, CD4+ and CD8+ T cells | Transcriptome profile of CD8+ T cells resembling an exhausted signature is correlated with good outcome in autoimmunity such as in AAV. | n.a. |
| O’Sullivan (89) | MPO-AAV, 47 | Before treatment | MPO-AAV: mean 67, 32M 15F |  | n.a. | Tubulointerstitial macrophages, CD4+ and CD8+ T cells, and neutrophils correlated with low presenting eGFR. |
| Kerstein (97) | GPA, 20  HCs, 20 | Mean 35 m  Active: n=4  Remission: n=16 | GPA: mean 64, range 44-74) 16M 4F | Flow cytometry on WB.  RNA from isolated CD4+ and CD8+ T cells for  transcriptome analysis, GPA n=3, HCs n=3.  PBMCs stimulated with viral peptide, percentage of dextramer positive cells determined.  Proliferation by CFSE dilution. | Increased percentages of CD4+CD28-, CD8+CD28- and CD4+CD8+ cells in GPA.  Transcriptome analyses reveal antigen and cytokine impact on T cells in GPA.  CMV and EBV associated with CD28- T cells in GPA. | n.a. |
| Chang (112) | Mouse model |  |  | Anti-MPO glomerulonephritis mouse model | n.a. | CD8+ T cell depletion led to less injury.  Pathogenic MPO-specific CD8+ T cells in a mouse model mediated by MPO-specific CD4+ T cells led to more severe disease.  Transfer without CD4+ T cells mediated injury when MPO was transferred in glomeruli. |
| Kidder (90) | AAV, 38 | Before treatment  MPA: n=24  GPA: n=14 | AAV: median 66, range 25-76. | IHC on renal biopsies of macrophages and T cells | n.a. | Interstitial macrophages and CD8+ T cells were correlated (weakly) with low renal function. |
| Chen (113) | Mouse model |  |  |  | n.a. | CD8+ T cells infiltrated glomeruli and were in direct contact with EGFP+ podocytes when there was disruption of Bowman’s Capsule. |

* WB: whole blood. APCs: antigen presenting cells. PBMCS: peripheral blood mononuclear cells. IF: immunofluorescence. IHC: immune histochemistry. PCR: polymerase chain reaction. PMN: polymorphonuclear neutrophils

**n.a. : not applicable
